# One and only SNARC? Spatial-Numerical Associations are not fully flexible and depend on both relative and absolute number magnitude

**DOI:** 10.1098/rsos.241585

**Published:** 2025-01-08

**Authors:** Lilly Roth, John Caffier, Ulf-Dietrich Reips, Hans-Christoph Nuerk, Annika Tave Overlander, Krzysztof Cipora

**Affiliations:** ^1^ Department of Psychology, University of Tübingen, Tübingen, Germany; ^2^ Department of Psychology, University of Konstanz, Konstanz, Germany; ^3^ LEAD Graduate School & Research Network, University of Tübingen, Tübingen, Germany; ^4^ German Center for Mental Health (DZPG), Mannheim, Germany; ^5^ Centre for Mathematical Cognition, Loughborough University, Loughborough, UK

**Keywords:** Spatial-Numerical Associations, SNARC effect, mental number line, replication, flexibility

## Abstract

Numbers are associated with space, but it is unclear how flexible these associations are. We investigated whether the SNARC effect (Spatial-Numerical Association of Response Codes; Dehaene *et al*. 1993 *J. Exp. Psychol*. **122**, 371–396. (doi:10.1037/0096-3445.122.3.371); i.e. faster responses to small/large number magnitude with the left/right hand, respectively) is fully flexible (depending only on relative magnitude within a stimulus set) or not (depending on absolute magnitude as well). Evidence for relative-magnitude dependency came from studies observing that numbers 4 and 5 were associated with the right in a 0–5 range but with the left in a 4–9 range (Dehaene *et al*. 1993; Fias *et al*. 1996 *Math. Cogn*. **2**, 95–110 (doi:10.1080/135467996387552). Within this Registered Report, we conducted two online experiments running Bayesian analyses with optional recruitment stopping at moderate evidence (BF_10_ above 3 or below 1/3). Experiment 1 (*n* = 200) replicated relative-magnitude dependency using the original stimuli. However, Experiment 2 (*n* = 300) additionally demonstrated absolute-magnitude dependency, while considering recent advances in SNARC research using 1–5 excluding 3 and 4–8 excluding 6. In contrast to the frequently perpetuated notion of fully flexible Spatial-Numerical Associations, some fixed relation to absolute magnitude prevails. These findings have important consequences for understanding how Spatial-Numerical Associations might support numerical processing.

## 1. Introduction

Numbers are highly relevant in everyday life. Therefore, much research has been devoted to understanding how we process and represent them in our minds. Interestingly, various aspects of numerical information such as cardinality and ordinality are systematically associated with different aspects of space such as extensions or directions [1–3]. This broad range of phenomena is referred to under the umbrella term Spatial-Numerical Associations (SNAs) [4,5]. Investigating these associations is fundamental for models of number representation and—considering the bigger picture—of human cognition.

The hallmark directional SNA is the Spatial-Numerical Association of Response Codes (SNARC) effect, which denotes that in left-to-right reading cultures, participants respond faster to small/large magnitude numbers on the left/right side, respectively [6]. Interestingly, the SNARC effect can be observed in a parity judgement task, in which the magnitude of the numbers is not task-relevant. This effect has been replicated using different modalities, set-ups and tasks (see [7] for an online replication; [5,8] for a recent review; [9] for a meta-analysis). The SNARC effect is typically quantified using the repeated-measures regression originally proposed by Lorch & Myers [10] and applied to the SNARC effect by Fias *et al.* [8]. In the first step mean differences in reaction times (RTs) between the right and left hand (dRTs) are regressed on numerical magnitude for each participant separately. A negative slope indicates an increasing right-hand advantage with increasing number magnitude (the more negative the so-called SNARC slope, the stronger the SNARC effect). Subsequently, to check for the SNARC effect at the group level, individual SNARC slopes are tested against zero with a one-sample *t*‐test.

Interestingly, several studies have documented that the SNARC effect is not fixed but might be prone to several types of manipulation ([11] for a taxonomy), for instance, changing the number range of the used stimuli, which has been classified as representational, intra-experimental manipulation. The spatial mental number representation seems to be adapted to fit the task at hand. In this work, we focus on the extent to which the SNARC effect flexibly adjusts to the specific range of the numbers being used in the task set.

### 1.1. Relative-magnitude (RM) dependency of the SNARC effect

The seminal paper by Dehaene *et al*. [6] has already demonstrated in its Experiment 3 that the SNARC effect depends on the relative rather than the absolute magnitude (AM) of numbers. They found the SNARC effect in two different numerical intervals ranging from 0 to 5 and from 4 to 9. In the lower interval, responses to numbers 4 and 5 were faster with the right hand than with the left (typical response pattern for large numbers) and right-hand responses to these numbers were faster than right-hand responses to lower numbers. In contrast, in the higher interval, responses to these numbers were faster with the left hand than with the right (typical response pattern for small numbers) and left-hand responses to these numbers were faster than left-hand responses to higher numbers. This finding was replicated by Fias *et al*. [8] (Experiment 1). It suggests that the SNARC effect dynamically adapts to the current task set (i.e. numbers being used) and is determined by the RM of the number within the set rather than its AM. We refer to this claim about the SNARC effect as *RM dependency*.

RM dependency is considered as one of the crucial features of the SNARC effect and has been taken for granted since these early findings. The results of Dehaene *et al*.’s [6] and Fias *et al*.’s [8] experiments are widely cited as an argument for the SNARC effect being dependent on the given number range (e.g. by [12–17]). The RM dependency of the SNARC effect has been demonstrated by several other studies even going beyond a basic set-up comprising judgements on single-digit numbers. For instance, Tlauka [18] found a SNARC effect both when using the two numbers, 1 and 100, and when using the two numbers 100 and 900. The number 100 was associated with the right/left when it was the larger/smaller of the two numbers, respectively. Ben Nathan *et al*. [19] went even further, showing that the SNARC effect is not only RM dependent on the task level but built up on a trial-to-trial basis. They found the right- and left-key response speed advantages in magnitude judgement tasks to depend on the RM in comparison to the ever-changing reference number. What is more, evidence for RM dependency of SNARC-like effects goes beyond numerical stimuli. Wühr & Richter [20] found a SNARC-like effect (association of physically smaller/larger stimuli with the left/right, respectively) to depend on relative rather than absolute stimulus size.

Importantly, RM dependency has also been used as a methodological tool to show that a spatial-numerical phenomenon is in fact the SNARC effect. For instance, Rugani *et al*. [21], Di Giorgio *et al*. [22] and Giurfa *et al*. [23] showed RM dependency to claim that a certain effect they observed in newly hatched chickens, in newborn children and in honeybees is of the same nature as the SNARC effect. To sum up, there is evidence for the RM dependency of the SNARC effect in various tasks and set-ups, and it has even been used to validate SNAs.

### 1.2. RM dependency in the light of number-representation models

RM dependency fits well with most theoretical accounts of number representation. The seminal work of Restle [24] outlining the mental number line (MNL) account, which has been proposed as the first explanation for the SNARC effect [6], postulates that the MNL is flexible and dynamically adapts to the task demands. In line with this, Pinhas *et al*. [17] claim that the resolution of the MNL can be adjusted to the numerical context. The accounts of verbal–spatial coding [25] and polarity correspondence [26] are on the one hand in line with RM dependency, but on the other hand, they do not make clear statements about RM being the *only* decisive factor determining the SNARC effect. Crucially, both accounts assume that long-term number representations underlie the SNARC effect, which hardly justifies the SNARC effect’s flexibility [15,27]. The working memory account [28,29] originally claimed that the SNARC effect does not rely on long-term number representations but is instead constructed during task execution, which speaks in favour of pure RM dependency. However, Ginsburg *et al*. [14] and Koch *et al*. [30] argue that short-term number representations do not always fully overrule long-term number representations. This idea has been incorporated in the hybrid account proposed by van Dijck *et al*. [27] as well, and it allows the coexistence of RM dependency and dependency of the SNARC effect on AM (henceforth *AM dependency*). Furthermore, concurrent RM dependency and AM dependency would also be in line with the idea that multiple number representations and multiple spatial reference frames can be activated and operated simultaneously [31]. To conclude, the assumption that AM plays no role can hardly be derived from theoretical accounts of the SNARC effect.

### 1.3. Hints towards AM dependency of the SNARC effect

In addition to the prominent claims on the RM dependency of the SNARC effect, the literature also provides hints towards an AM dependency of the SNARC effect. It is important to note that AM dependency can, on the one hand, influence the strength of the SNARC effect (reflected by the SNARC slope), and on the other, the location of numbers on the MNL in absolute terms (reflected by the intercept of the regression line and by dRTs of critical numbers that are part of both number ranges). Crucially, the SNARC effect seemed to be stronger in the lower than in the higher number range in both initial studies demonstrating the RM dependency (−20.1 ms versus −10.9 ms in Dehaene *et al*. [6]; and −10.18 versus −7.19 ms in Fias *et al*. [8]), suggesting AM dependency as well. In Fias *et al*.’s (1996) results, the observed slope difference had approximately an effect size of Cohen’s *d* = 0.16 (i.e. the slope difference of 2.99 divided by the standard deviation [SD] for this slope difference of 18.34 ms, which has been calculated with SD = 15.1 ms and SD = 11.2 ms for the lower and higher number ranges, assuming a rather conservative correlation between them of *r* = 0.05, which corresponds to the correlation we have observed in our previous colour judgement tasks, see Roth *et al*. [32], where we also found a stronger SNARC effect in the lower than in the higher half of the stimulus set ranging from 1 to 9). Moreover, the results pointed towards an overall shift of small/large numbers to the left/right on the MNL, respectively, since the smallest-number intercept (i.e. the predicted dRT for the smallest number magnitude of the range, which was 0/4 in the lower/higher range, respectively) was larger in the lower than in the higher range (37.52 ms versus 14.03 ms in Dehaene *et al*. [6], and 15.43 ms versus 8.82 ms in Fias *et al*. [8]). However, the mean-number intercepts (i.e. the predicted dRT for the mean number magnitude of the range, which was 2.5/6.5 in the lower/higher range, respectively) did not differ much in Fias *et al*.’s results (−10.02 ms versus −9.16 ms). In Dehaene *et al*.’s results, this intercept seemed to be smaller in the higher number range, but it cannot be calculated exactly based on the data reported in the paper.

### 1.4. Methodological limitations of the two initial studies demonstrating RM dependency

Even if we use the two original studies as guidance for further investigations, their findings are not reliable because of several important limitations regarding the design and the interpretation of the results. Both Dehaene *et al*. [6] and Fias *et al*. [8] found a significant two-way interaction of response side (left versus right) and magnitude (small versus medium versus large). Apart from the repeated-measures regression approach, the SNARC effect can also be quantified as a two-way interaction of response side and magnitude (for methodological considerations, see Fias *et al*. [8]) or as linear contrast in an ANOVA [33]. However, the three-way interaction of response side and magnitude with interval (0–5 versus 4–9) remained non-significant in both studies. In Fias *et al*.’s [8] additional repeated-measures regression, the resulting SNARC slopes differed significantly from zero in both intervals in a one-sample *t*‐test, and the difference in SNARC slopes between both intervals remained non-significant in a paired *t*‐test. Crucially, the strong conclusion of pure RM dependency that has been derived from these null results is dangerously close to mistaking the absence of evidence for evidence of absence. Importantly, no Bayesian analysis was conducted to test whether the null results supported the null hypothesis (and it is not possible to run a post-hoc Bayesian analysis due to the lack of a report of the exact *t*-statistic). What is more, neither Dehaene *et al*. [6] nor Fias *et al*. [8] tested whether the dRT pattern for the same number differed significantly between number ranges—even if the right-hand advantage (reflected by negative dRTs) for numbers 4 and 5 in the range from 0 to 5 and the left-hand advantage (reflected by positive dRTs) for these numbers in the range from 4 to 9 are often cited. Also, the smallest-number intercepts and the mean-number intercepts were not compared between ranges.

Moreover, the design was most likely underpowered for the relevant statistical comparisons in both studies (see below for calculations). On the one hand, this was due to the relatively low sample sizes (*n* = 12 in Dehaene *et al*. [6] and *n* = 24 in Fias *et al*. [8]). On the other, only 15 repetitions per experimental cell (i.e. per number magnitude and response-key assignment) were used. Later methodological studies proposed to use at least 20 repetitions and 20 participants to detect the SNARC effect, and even more repetitions and participants to detect differences in the size of the SNARC effect [34]. Following the *effect-size sensitivity approach* [35], we have run power calculations to determine SNARC slope differences between the two number ranges that are detectable in a *t*‐test for two dependent samples at different adequate power levels (adapting Monte-Carlo simulations by Wickelmaier [36]). For the sample size used by Fias *et al*. [8] and with the SD they observed, our calculations revealed that at power levels of 0.80, 0.90 and 0.95, only SNARC slope differences between the two number ranges of minimum 11.0 (*d* = 0.60), 12.7 (*d* = 0.69) and 14.1 ms (*d* = 0.77) could have been detected, respectively. Note that we ran these calculations within the frequentist framework, which corresponds to the data analysis by Fias *et al*. (for calculations in both the frequentist and the Bayesian framework, see ‘PCI Registered Report Materials’ at [37], created using the R packages *rmarkdown* by Allaire *et al*. [38]; *knitr* by Xie [39], and *BayesFactor* by Morey *et al*. [40]). However, such differences in SNARC slopes are very unlikely, even in the case of AM dependency, because they would be larger than the typically observed SNARC slopes themselves. Because of the lack of related information in Dehaene *et al*.’s (1993) paper, we were not able to run such power calculations for their results; but because their sample was even smaller, they could have detected only even larger differences.

Moreover, the stimuli used in both studies (0, 1, 2, 3, 4, 5 and 4, 5, 6, 7, 8, 9) lead to two problems. First, the average number magnitude in both number ranges is larger for odd than for even numbers (3 versus 2 in the lower and 7 versus 6 in the higher number range). This can lead to a confound with the MARC (Linguistic Markedness of Response Codes) effect that denotes a left-/right-hand advantage when responding to odd/even numbers, respectively [41]. Such a confound may decrease the SNARC effect [33,42]. The association of small/large numbers to the left/right side, respectively, should be weaker if small/large numbers are more often even/odd, respectively. More recent studies have addressed this issue by using stimuli sets in which number magnitude and contrast-coded parity are orthogonal (e.g. [7]). Typically, it is done by using the number set 1, 2, 3, 4, 6, 7, 8 and 9, which importantly also excludes 0.

Second, using the number 0 is problematic due to its special status shown in several studies: reading time for 0 is significantly longer than for any other single-digit number and is not predicted by factors determining the reading time of other single-digit numbers [43]. Nuerk *et al*. [41] and Nieder [44] provide further empirical evidence that 0 may not be represented on the MNL along with other numbers (but see Pinhas & Tzelgov [45], for another conclusion). Additionally, quite often participants have problems understanding the parity status of 0 [46]. Using 0 also turned out problematic in SNARC studies: the RTs and dRTs for the number 0 do not strongly correlate with the RTs and dRTs of other even numbers [41]. Later studies on the SNARC effect excluded 0 from the stimuli set (e.g. [13,25,47–49]). Ultimately, both the parity status and the presence of 0 might have confounded the results of the previous studies (see table 1). Therefore, in addition to the replication that we conducted as close as possible to the original studies by Dehaene *et al*. [6] and Fias *et al*. [8], we also ran a conceptual replication using suitable stimulus sets to disentangle these potential confounds and tackle all the above-mentioned limitations.

**Table 1.** Stimulus sets and their characteristics.

Experiment 1 (close replication: number ranges used by [6,8])				Experiment 2 (conceptual replication)			
lower range		higher range		lower range		higher range	
absolute magnitude predictor	contrast-coded parity predictor	absolute magnitude predictor	contrast-coded parity predictor	absolute magnitude predictor	contrast-coded parity predictor	absolute magnitude predictor	contrast-coded parity predictor
0	+0.5	**4**	+0.5	1	−0.5	**4**	+0.5
1	−0.5	**5**	−0.5	2	+0.5	**5**	−0.5
2	+0.5	6	+0.5	**4**	+0.5	7	−0.5
3	−0.5	7	−0.5	**5**	−0.5	8	+0.5
**4**	+0.5	8	+0.5	—	—	—	—
**5**	−0.5	9	−0.5	—	—	—	—
mean number magnitude depending on number parity:							
*M*_even_ = 2 *M*_odd_ = 3		*M*_even_ = 6 *M*_odd_ = 7		*M*_even_ = 3 *M*_odd_ = 3		*M*_even_ = 6 *M*_odd_ = 6	
correlation between number magnitude and number parity:							
*r* = –0.293				*r* = 0			

This table lists all stimuli used in the two experiments. It shows the confound between number parity and number magnitude in both number ranges of Experiment 1 and illustrates how we avoided it in both number ranges of Experiment 2, such that number parity and number magnitude were uncorrelated (i.e. they were orthogonal to each other as predictors in regression models). Number parity was contrast-coded with −0.5 for odd and +0.5 for even numbers when measuring the MARC effect. The number 0 was included in Experiment 1, but not used in the conceptual replication in Experiment 2 because of its special features and irregular mental representation (as outlined in the Introduction). The numbers 4 and 5, which are written in bold in the table, were present in each of the number ranges.

### 1.5. The SNARC effect operating on two reference frames at once

As we laid out so far, there is a general tendency to interpret the SNARC effect as entirely flexible based on the findings of RM dependency and on the inference-statistical null effects concerning AM dependency (in underpowered studies). However, the SNARC effect could be operating concurrently in both relative and absolute terms. Indeed, one of us has proposed that the SNARC effect operates on multiple number lines [31]. However, that paper is not about whether the SNARC effect operates on multiple number lines in terms of RM dependency and AM dependency, but instead, it used two-digit numbers as stimuli to see whether separate number lines are activated for decade and unit numbers. The operations on different number ranges are for decade and unit digits of one two-digit number (i.e. the same number, but different digits of its decomposition). Thus, the paper by Weis *et al*. provides the principal account that the SNARC effect could operate on multiple reference frames at once. The current study goes beyond their findings because it seeks to demonstrate that both RM-dependent and AM-dependent spatial mappings are concurrently present in the same digit.

### 1.6. The current study

In this study, we aim to answer the question whether the SNARC effect depends only on RM or whether AM plays a role as well. Crucially, in contrast to previous literature about the flexibility of the SNARC effect, we differentiate between two concepts that can be affected by RM dependency and AM dependency:

- On the one hand, the number mapping on the MNL (e.g. dRT for number 4) may be different depending on the experimental set-up. In our set-up, it can be RM-dependent (i.e. depending on the position on the used range, e.g. position 5 for range 0–5, or 1 for range 4–9), AM dependent (i.e. depending on the magnitude, e.g. 4), or both at the same time.
- On the other hand, the strength of the SNARC effect relies on the relative increase of right-hand advantage per increase in magnitude (i.e. the steepness of the SNARC slopes, e.g. −5 ms per number or −10 ms per number) and these slopes can differ between ranges.

For a more detailed and complex elaboration of six possible scenarios combining different parameters of (i) and (ii), see electronic supplementary material, figures S1 and S2 (see ‘PCI Stage 2 Registered Report Materials’ at [37].

To answer the research question, we first replicated Experiment 3 by Dehaene *et al*. [6], which has also been replicated in Experiment 1 by Fias *et al*. [8], using the original number ranges from 0 to 5 and from 4 to 9. Second, we conducted a conceptual replication to address confounds due to the unequal distribution of odd and even numbers and due to the presence of 0 in both stimuli sets, using the number ranges 1 to 5 (excluding 3) and 4 to 8 (excluding 6). The middle number of the range is also excluded in most SNARC studies using the typical set from 1 to 9. This way, the critical numbers that appear in both ranges were the same in both experiments, namely 4 and 5. Table 1 gives an overview of the used number ranges and of confounds between number parity and number magnitude in Experiment 1 that were avoided in Experiment 2.

In both of our replication experiments, high statistical power was obtained by testing much larger samples than Dehaene *et al*. [6] and Fias *et al*. [8] and by increasing the number of repetitions per experimental cell from 15 to 25. To be able to quantify evidence both for differences between number ranges and lack thereof, we applied the Bayesian instead of frequentist approach in statistical analysis. For the interpretation of different values for the Bayes factors, we followed the recommendations by Dienes [50]: a BF_10_ greater than 3 or 10 was treated as moderate or strong evidence for the alternative hypothesis, while a BF_10_ smaller than 1/3 or 1/10 was treated as moderate or strong evidence for the null hypothesis, respectively.

Online experiments offer the possibility to collect data from large samples and therefore reach high statistical power [51,52]. The SNARC effect has been successfully replicated in online settings (e.g. [7,30,49]). The measurement in the online set-up showed a similar reliability and magnitude compared to the SNARC effect that is typically observed in lab studies. Further, it seems to be valid as regards the correlations of the SNARC effect with mean RTs and SD of RTs, which are similar to lab studies.

In this study, we expected to replicate the findings by Dehaene *et al*. [6] and by Fias *et al*. [8] as concerns RM dependency. However, we also expected to find evidence towards AM dependency of the number mapping on the MNL and of the strength of the SNARC effect. Previous studies have indicated tendencies that cannot be explained by RM dependency alone. Thus, we hypothesized the following:

1. A SNARC effect in both (a) the lower and (b) the higher number ranges in each experiment. (a) The SNARC effect in the lower range served as a manipulation check and was considered as a prerequisite for testing hypotheses 2 and 3 in each experiment. Both (a) and (b) aimed at replicating the results by Dehaene *et al*. [6] and Fias *et al*. [8].
2. Both (a) RM dependency and (b) AM dependency of the number mapping on the MNL, such that small/large numbers in relative and absolute terms are shifted towards the left/right, respectively. (a) RM dependency is reflected by dRTs for the same critical numbers (i.e. 4 and 5) differing between ranges, showing that the MNL adapts flexibly and relative to the range. (b) AM dependency is reflected by dRTs for these critical numbers being equal between ranges, and by dRTs for the smallest number (Experiment 1: 0 in the 0–5 range versus 4 in the 4–9 range; Experiment 2: 1 in the 1–5 range [excluding 3] versus 4 in the 4–8 range [excluding 6]) differing between ranges. AM dependency means that small/large numbers are shifted to the left/right on the MNL, although they are in exactly the same position within their range, but differ in terms of AM.
3. AM dependency of the strength of the SNARC effect, such that it is stronger in the lower than in the higher ranges. This is reflected by steeper (i.e. more negative) SNARC slopes in the lower than in the higher ranges, which was descriptively observed in the two seminal studies by Dehaene *et al*. [6] and Fias *et al*. [8].

## 2. Materials and method

This study has been approved by the ethics committee of the University of Tübingen’s Department of Psychology. The Stage-1 Registered Report received in-principle acceptance by the Peer Community In (PCI) on 3 December 2023, and the Stage-2 Registered Report received their final recommendation on 6 September 2024 (https://doi.org/10.24072/pci.rr.100808).

### 2.1. Sample size considerations

For this study, we defined Cohen’s *d* = 0.15 as the minimal effect size of interest, because the most crucial aim of the present study was to find out whether AM dependency of the strength of the SNARC effect exists or not (hypothesis 3). By choosing this minimal effect size of interest, we were able to find evidence for or against the SNARC slope differences between number ranges that had been descriptively reported in the original studies that we wished to replicate. Due to the lack of a report of SD, it was not possible to calculate Cohen’s *d* for the slope difference of 9.2 ms found by Dehaene *et al*. [6], but the slope difference of 2.99 ms with its SD of 18.34 ms found by Fias *et al*. [8] corresponds to an effect size of *d* = 0.16. Note that in the two original studies, the symmetric confidence intervals for these estimates must also include at least the double slope difference and effect size due to their non-significance. Hence, in case of AM dependency of the strength of the SNARC effect, the true effect size might in fact be larger than *d* = 0.15. This sample size estimation was also valid for testing hypotheses 1 and 2, which required one-sample *t*-tests. The reason was that an effect smaller than *d* = 0.15 would not be meaningful for the SNARC effect in the lower (hypothesis 1a) or higher (hypothesis 1b) number range, or for RM dependency (hypothesis 2a) and AM dependency (hypothesis 2b) of the number mapping on the MNL either. Similarly, the chosen maximal sample size was large enough to find at least moderate evidence in case these hypotheses are false.

To ensure a probability of 0.90 for finding at least moderate evidence for a true underlying effect (i.e. BF_10_ above 3, according to Dienes [50] with a minimally relevant effect size of Cohen’s *d* = 0.15 in one-sample or paired *t*-tests, the sample needed to consist of 800 participants in each experiment (for power calculations, see ‘PCI Registered Report Materials’ at [37]. The sample size of 800 participants was required for a proportion of at least 0.90 Bayesian *t*-tests to yield a BF_10_ above 3 when 5000 samples of SNARC slope differences randomly drawn from a normal distribution around the minimally relevant effect size of *d* = 0.15 were simulated (for a similar approach, see [53]). Following the same procedure, we found that the sample needed to consist of 180 participants to ensure a probability of 0.90 for finding at least moderate evidence against an effect that is truly absent (i.e. BF_10_ below 1/3 for *d* = 0, according to Dienes [50]). Note that the sample size of 180 was smaller than the initial sample size of 200 that was collected in the ‘Sequential Bayes Factor with maximal n’ (SBF + maxN) approach as described by Schönbrodt & Wagenmakers [54] (see explanation below). For these calculations, we used SD = 15.1 ms and SD = 11.2 ms for the lower and higher number ranges, as reported by Fias *et al*. [8], although the SD in our previous colour judgement experiments were only SD = 4.2 ms and SD = 3.9 ms [32]. Hence, our calculations were rather conservative, and the probability of finding evidence for a true underlying effect thus was most probably even higher. While in the frequentist framework, low error type II rates (and high statistical power) need to be achieved, in the Bayesian framework, a low probability of misleading evidence for the null hypothesis in case of a true underlying effect and a high probability of finding evidence for a true underlying effect needs to be ensured. To achieve the same probability for error type II and misleading evidence, Bayesian *t*-tests (using the default *r*-scale of 0.707 as uninformed prior in the Cauchy distribution) require larger samples as compared to frequentist *t*-tests [53].

Importantly, as we ran Bayesian instead of frequentist analyses, we made use of the SBF + maxN approach and defined an optional stopping threshold to make our data collection more efficient. Namely, we used moderate evidence in favour of all hypotheses (BF_10_ > 3) or against them (BF_10_ < 1/3) as thresholds. More precisely, for each experiment, we first recruited 200 participants (i.e. complete individual datasets) and computed the BF_10_ for the SNARC effect in lower (hypothesis 1a) and higher (hypothesis 1b) number ranges, for the shift of critical small/large numbers in both relative (hypothesis 2a) and absolute (hypothesis 2b) terms towards the left/right, respectively, and for the SNARC slope difference between ranges (hypothesis 3). As long as the BF_10_ did not reach any of the two thresholds for all hypotheses, we collected another 20 complete individual datasets and recalculated the BF_10_. If no threshold had been reached with our maximal sample size of 800 participants (that is required for obtaining at least moderate evidence for a true underlying minimally relevant effect with a probability of at least 0.90, as explained above), we would have stopped the sequential recruiting of participants in any case.

### 2.2. Participants

For each experiment, adults were recruited via the recruiting platform Prolific. To comply with our ethics proposal, they had to be at least 18 years old, and because of possible age differences in RTs, we set the maximum age to 40 years. As the experiments were conducted in English, participation was only possible for native English speakers (as per Prolific’s screening based on self-reports). Participation took approximately 20 min and was compensated with £5 (partial payment for partial participation).

### 2.3. Design and experimental task

In the parity judgement task with binary response-key set-up, participants had to indicate as fast and as accurately as possible whether the number presented on the screen was odd or even. The parity judgement task is widely used in numerical cognition and the standard task to investigate the SNARC effect (see [5] for a review and Wood et al., 2008 [9] for a meta-analysis). We assigned participants randomly to one of our two experiments. In Experiment 1 (close replication of Dehaene *et al*. and Fias *et al*. [6,8]), the numbers from 0 to 5 were used in the lower number range and the numbers from 4 to 9 in the higher number range. In Experiment 2 (conceptual replication), the numbers from 1 to 5 (excluding 3) were used in the lower number range and the numbers from 4 to 8 (excluding 6) in the higher number range, eliminating confounds between number parity and number magnitude (see table 1) and special influences of the number 0.

**Figure 1.**
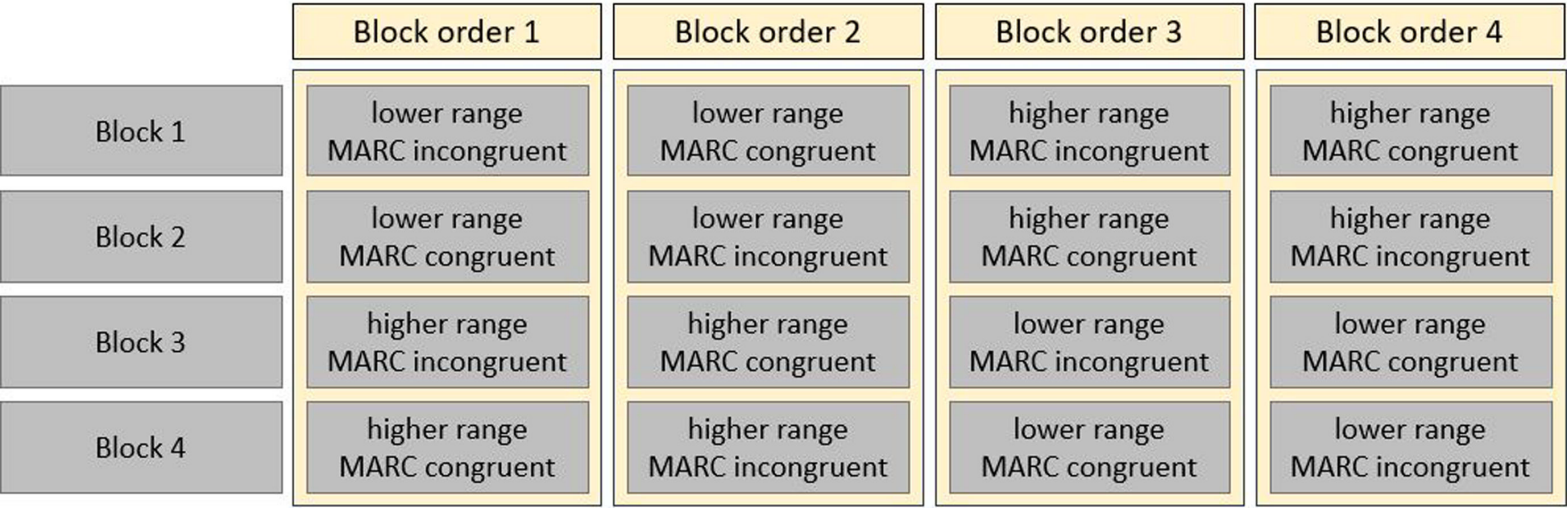
Counterbalancing block orders in experiments 1 and 2. *Note*. This figure shows the four block orders resulting from the combination of range (lower range versus higher range) and response-key assignment (MARC congruent, i.e. odd-left and even-right, versus MARC incongruent, i.e. even-left and odd-right). Each block was preceded by two repetitions per number as practice trials (12 trials for Experiment 1 and eight trials in Experiment 2), consisted of 25 repetitions per number as experimental trials (150 trials for Experiment 1 and 100 trials in Experiment 2) and was followed by a break.

**Figure 2.**
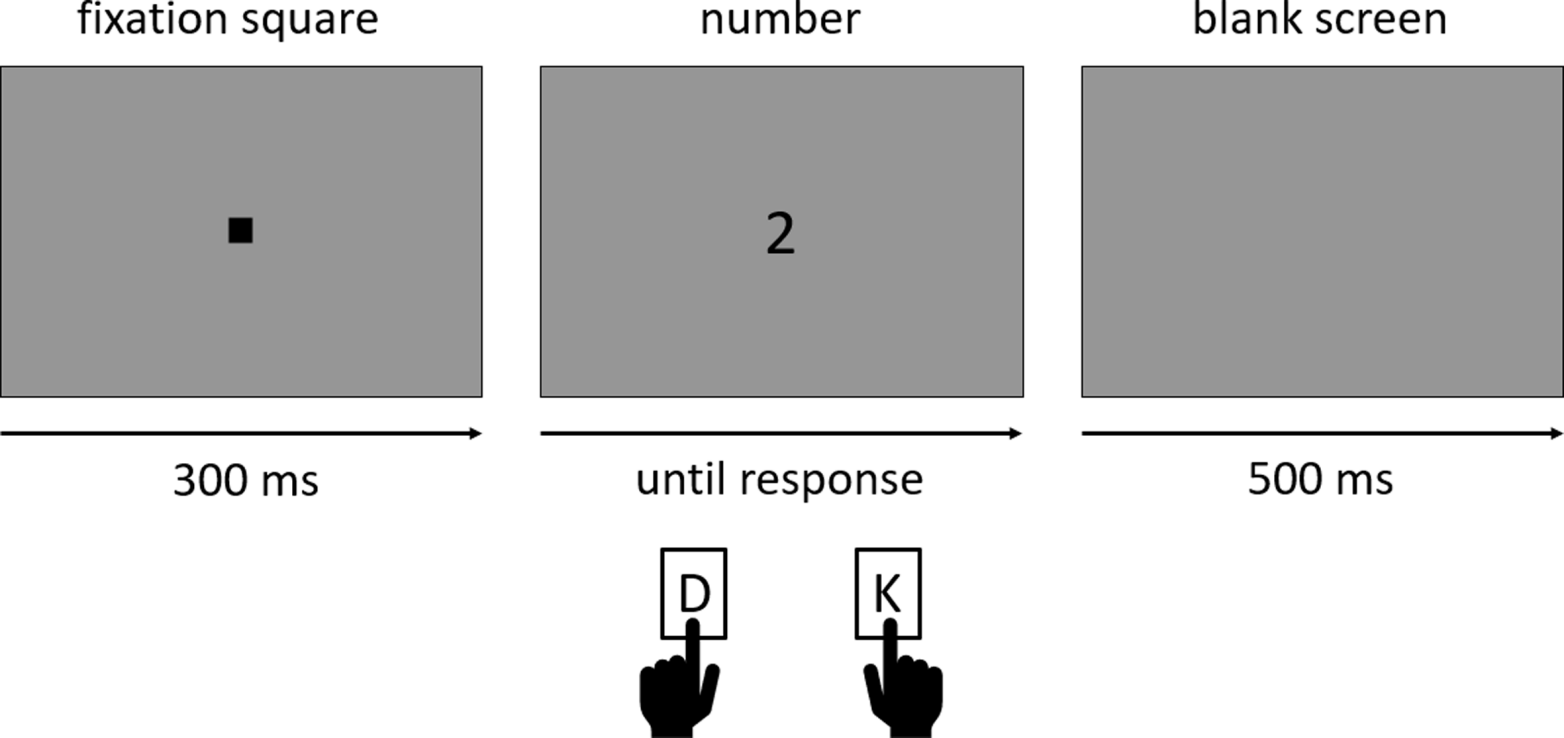
Time course of an exemplary trial.

In both experiments, we used 25 repetitions per number magnitude in each number range (lower versus higher) and each response-key assignment (MARC congruent, i.e. left-hand responses to odd and right-hand responses to even numbers, versus MARC incongruent, i.e. right-hand responses to odd and left-hand responses to even numbers). This lead to a total of 600 trials for Experiment 1 and 400 trials for Experiment 2. In each experiment, the trials were equally divided into four blocks (one per combination of number range and response-key assignment), with a break of minimum 30 s between them. Participants were randomly assigned to one of four block orders of congruency and number range (see figure 1). The order of stimulus presentation within blocks was fully randomized. Each trial started with a square (extended ASCII 254 with a font size 72px) as an eye fixation point (300 ms). Then the number (Open Sans font, size 72px) was presented until a response was given. A blank screen (500 ms) concluded the trial. Stimuli, as well as fixation squares, were presented in black (0, 0, 0 in RGB notation), while the background remained grey (150, 150, 150 in RGB notation). The time course of an exemplary trial is illustrated in figure 2. Each block was preceded by a short practice session, in which each number was presented twice (i.e. 12 practice trials before each block in Experiment 1 and eight practice trials before each block in Experiment 2). Accuracy feedback appeared during practice sessions only.

### 2.4. Procedure

The experiments were set-up with WEXTOR (https://wextor.eu; [55]) in its HTML and JavaScript framework and adapted (see demo versions for Experiment 1 at https://exp.wextor.eu/esnarc/flex/?demo&e1 and for Experiment 2 at https://exp.wextor.eu/esnarc/flex/?demo&e2). Our previous experiments [32] have demonstrated that this software is suitable for detecting the SNARC effect in an online set-up. At the very beginning of the experiment, a seriousness check [56,57,58] was applied and participants were asked whether they wanted to participate seriously. Participants were asked to take part only if they gave their informed consent if they were using a desktop computer or laptop and if they were between 18 and 40 years old. Then, participants were asked to provide basic demographic data such as age, gender (*man*, *woman*, *other*), first native language (English and potentially others), handedness (*right-handed*, *left-handed*, *ambidextrous*) and finger-counting habits (starting hand: *left hand*, *right hand*, *does not know or no preference*; and stability: *always*, *usually*, *does not know or no preference*). For each of the above-mentioned questions, we also provided the option ‘I prefer not to answer’ to respect some participants’ unwillingness to share information with us and to not force them to choose any option that might not reflect the truth [59,60]. Note that in earlier studies, only very few participants chose this option in any of the above-mentioned questions. Next, if not already the case for the default response keys D and K, participants could choose response keys for the experimental task which were to be located in the same row and about one hand width apart from each other on their keyboard. Then, instructions were displayed before the first block of the experimental task started with its practice trials. For instance, the instructions were as follows for the block with the lower number range in Experiment 1 (only numbers and response-to-key assignments are replaced for the higher number range or for Experiment 2): ‘In our experiment, your task is to distinguish the parity of numbers, that is, to decide whether a number is even or odd. For this, please place the index finger of your left hand on the [D] key and the index finger of your right hand on the [K] key on your keyboard. In each run, a black square will appear in the centre of the screen. Please look at this square. It will soon be replaced by either an even or an odd number. If the number is even (0, 2, 4), press [D]. If the number is odd (1, 3, 5), press [K]. Please answer as quickly and as accurately as possible’.

After completion of the whole experimental task, participants were asked to self-rate their math skills compared to people of their age on a visual analogue scale from *very bad* to *very good* operationalized as 0 to 400 [61]. Next, data quality was assessed by asking participants how they would describe their environment during participation (*silent*, *very quiet*, *fairly quiet*, *fairly noisy*, *very noisy* or *extremely noisy),* whether there were any major distractions during participation (*none*, *one* or *multiple*) and whether there were any difficulties during participation (*yes* or *no*, text field for comments). Moreover, we asked participants whether they had used their left index finger for the left response key and their right index finger for the right response key throughout the experiment (*yes*, *partly* or *no*). Participants were provided with a completion code for Prolific and contact information of our research team. To prevent search engine bots (e.g. Googlebot) from submitting data on our experiment, we equipped the experiment materials with a standardized ‘noindex, nofollow’ meta tag, which prompts search engine bots not to index the experiment pages and also not to visit subsequent pages [62, p. 379]. Further, we restricted participation to devices over 600-pixel screen width (i.e. no smartphones). In addition, to exclude multiple submissions by the same participants, submissions from the same IP addresses were not permitted.

### 2.5. Data preprocessing

We used the same analysis pipeline as in another of our studies, except for not applying any colour vision check [32]. This pipeline is similar to that used by Cipora, van Dijck, *et al*. [47] in an extensive re-analysis of 18 datasets and allowed us to reliably detect the SNARC effect. The preprocessing steps were applied in the exact order as they are listed in the following. Specifically, only datasets of participants who were between 18 and 40 years old and able to seriously participate were included. Individual datasets were excluded if participants described their environment as very/extremely noisy, if they reported multiple major distractions or if they reported that they were not using their left/right index finger for the left/right response key, respectively. As outlined by Reips [52] and Birnbaum [63], experimenters were recommended to control for potential multiple submissions from the same participants by checking their User-Agents (OS and browser information) and IP addresses.^1^ Regarding the data from all remaining participants, practice trials and incorrectly answered trials were not analysed. Only trials with RTs of minimum 200 ms were included in the analysis, because parity judgements faster than 200 ms are very unlikely and faster responses can therefore be treated as anticipations. Moreover, only trials with RTs of maximum of 1500 ms were included, because healthy educated adults should be capable of judging the parity status of single-digit numbers in less than 1500 ms, so that slower responses are unlikely to reflect only the mental process underlying parity judgement but instead might be caused by distractions. In the next step, further outliers were removed in an iterative trimming procedure for each participant separately, such that only RTs that are maximum 3 SD above or below the individual mean RT of all remaining trials were considered. This procedure allowed us to exclude RTs that were unlikely for each given participant and accounts for the right-skewed distribution of RTs, where the means would otherwise have been largely overestimated. After these exclusions at the trial level, only data of participants with at least 75% valid remaining trials (after excluding errors and outlier RTs) were included in the analysis at the participant level. Finally, only datasets of participants without any empty experimental cell (number magnitude per response side) in both number ranges were considered, because an empty cell would have caused a missing dRT, which in turn would have made the calculation of the SNARC slope problematic. Only complete individual datasets were included in the analysis (and none of the incomplete individual datasets fulfilled the inclusion criteria listed above).

### 2.6. Data analysis

All data analyses were performed in the statistical computing software R (version 4.3.3; [64]), using the R packages *BayesFactor* [40] *data.table* [65], *dplyr* [66], *GeneNet* [67], *ggplot2* [68], *neatStats* [69], *plyr* [70] and *tidyr* [71]. An overview of all research questions with corresponding hypotheses, the targeted sample size and planned analyses with a rationale, as well as the interpretations of potential outcomes and theoretical conclusions, is given in the Study Design Table (see table 2). Instead of frequentist analysis, we decided to take the Bayesian approach. For this, we determined the BF_10_ associated with the corresponding Bayesian *t*‐test to obtain evidence for both null and alternative hypotheses (using a default *r*-scale of 0.707 as uninformed prior using Cauchy distribution). More specifically, we calculated Bayesian *t*-tests and extracted the respective BF_10_. Considering a BF_10_ larger than 3 as evidence against the null hypothesis is less likely than rejecting a null hypothesis with a conventional significance level of *α* = 0.05 in the frequentist approach [72]. As explained above, we applied the SBF + maxN approach for sequential data analysis with optional stopping in case of at least moderate evidence for or against all hypotheses.

**Table 2.** This Study Design Table contains all research questions with corresponding hypotheses, the targeted sample size and planned analyses with a rationale, as well as the interpretations of potential outcomes and theoretical conclusions. All entries apply to both Experiment 1 (direct replication using 0–5 and 4–9) and Experiment 2 (conceptual replication using 1–5 excluding 3 and 4 to 8 excluding 6). The template for the Study Design Table was taken from PCI-RR and filled in before data collection started. Only the rightmost column, ‘bserved outcome’ has been added after data was collected and analyses were run.

Question	Hypothesis	Analysis plan	Interpretation given different outcomes	Theory that could be shown wrong by the outcomes	Observed outcome
Can a SNARC effect be observed in all number ranges?	*Hypothesis 1 (and manipulation check):* A robust SNARC effect is expected in both (a) the lower and (b) the higher number ranges, i.e. we expect to find at least moderate evidence for SNARC slopes (one per participant and per number range, calculated by regressing dRTs on number magnitude) to be smaller than zero in each number range. As the SNARC effect is very robust, especially for lower ranges and possibly stronger than in higher ranges, the SNARC effect in lower ranges (hypothesis 1a) will be used as manipulation check and prerequisite for following investigations (hypotheses 1b, 2a, 2b and 3).	Four regressions of dRTs on number magnitude followed by four two-sided Bayesian one-sample *t*-tests of SNARC slopes against zero (Experiment 1: 0–5 and 4–9; Experiment 2: 1–5 (excluding 3) and 4–8 (excluding 6)	Finding moderate or even strong evidence for a SNARC slope smaller than 0 in a Bayesian *t*‐test in each number range would provide evidence for a SNARC effect in both the lower (hypothesis 1a) and higher (hypothesis 1b) number ranges and be in line with results from previous studies (e.g. the two seminal studies by Dehaene *et al.* [6] and by Fias *et al*. [8]).	The SNARC effect in the parity judgement task has been shown in numerous studies using different number ranges within the interval from 0 to 9 (as in all scenarios, see electronic supplementary material, figures S1 and S2 and table S1: [37]. We therefore expect to find at least moderate evidence for it in all four number ranges. Finding at least moderate evidence against the SNARC in any of the four number ranges would be highly surprising, especially in the lower number ranges. Evidence against the SNARC effect in the higher ranges (hypothesis 1b) combined with evidence for the SNARC effect in the lower ranges (hypothesis 1a) would provide support for AM dependency of the strength of the SNARC effect (hypothesis 3).	**Experiment 1:** Strong evidence for a SNARC effect in the lower range with BF_10_ = 6956.04 (*d* = 0.38) and in the higher range with BF_10_ = 2.63 × 10^6^ (*d* = 0.47). **Experiment 2:** Strong evidence for a SNARC effect in the lower range with BF_10_ = 1.61 × 10^21^ (*d* = 0.71) and in the higher range with BF_10_ = 1.38 × 10^12^ (*d* = 0.52). **Summary:** Manipulation check successful in both experiments, replication of previous results, prerequisite for following investigations fulfilled.
Does the number mapping on the MNL depend on whether it is the lowest versus highest number in the current number range?	*Hypothesis 2a:* For the same critical number, a left-/right-hand advantage is expected when it is the lowest/highest number in the current number range, respectively. We hypothesize RM dependency (and possibly AM dependency as well, see hypothesis 2b) of the number mapping on the MNL.	Four two-sided paired Bayesian *t*-tests of dRTs for the same number in lower versus higher number range (i.e. for 4 and 5 in each experiment. (Note that this test will only be run in case we find at least moderate evidence for a SNARC effect in the lower number range of the respective experiment; see hypothesis 1a, which serves as a manipulation check.)	Finding moderate or even strong evidence for a different pattern for numbers that appear in both number ranges in the lower and the higher number range in a *t*‐test would provide evidence for RM dependency of the SNARC effect. Finding moderate or even strong evidence against a different dRT pattern would indicate AM dependency of the number mapping on the MNL.	Evidence for RM dependency would indicate flexibility of the MNL, such that its resolution adapts to the context and that RM plays a role for SNAs. However, this does not rule out the possibility that AM plays a role as well (see below). Evidence for AM dependency would indicate that the MNL is at least not fully flexible. Full RM dependency is illustrated in scenarios 1 and 4, full AM dependency is shown in scenarios 3 and 6 and a combination of both corresponds to scenarios 2 and 5 in electronic supplementary material, figures S1 and S2.	**Experiment 1:** Strong evidence for differences in dRTs for critical numbers between the ranges with BF_10_ = 2.57 × 10^4^ (*d* = 0.40) for number 4 and BF_10_ = 12.86 (*d* = 0.25) for number 5. **Experiment 2:** Strong evidence for differences in dRTs for critical numbers between the ranges with BF_10_ = 6.64 × 10^7^ (*d* = 0.42) for number 4 and BF_10_ = 64.64 (*d* = 0.24) for number 5 **Summary:** Evidence for RM dependency and thus flexibility of the number mapping on the MNL in both experiments.
Does the mapping of numbers on the MNL depend on whether they are small versus high numbers in absolute terms?	*Hypothesis 2b:* A left-/right-hand advantage could be observed for small/large numbers in absolute terms, respectively (on top of RM dependency, see hypothesis 2a). However, we cannot derive any clear hypothesis from the literature about whether dRTs are lower for the smallest number in a higher than in a lower range (as observed by Dehaene *et al*. [6], but not by Fias *et al*. [8].	Two two-sided paired Bayesian *t*-tests of smallest-number intercept in lower versus higher number range (one test per experiment). (Note that this test will only be run in case we find at least moderate evidence for a SNARC effect in the lower number range of the respective experiment; see hypothesis 1a, which serves as a manipulation check.)	Finding moderate or even strong evidence for different smallest-number intercepts in the lower and the higher number range in a Bayesian *t*‐test would indicate AM dependency of the number mapping on the MNL. Finding moderate or even strong evidence against different smallest-number intercepts would indicate RM dependency of the number mapping on the MNL.	Evidence for AM dependency would indicate that the MNL and the SNARC effect are not fully flexible and that AM plays a role for SNAs. However, this does not rule out the possibility that RM plays a role as well (see above). Evidence for RM dependency would indicate that the MNL is at least partly flexible.	**Experiment 1:** Strong evidence against a difference in smallest-number intercepts between the ranges with BF_10_ = 0.09 **Experiment 2:** Strong evidence for a difference in smallest-number intercepts between the ranges with BF_10_ = 546.98 (*d* = 0.27) **Summary:** Evidence against AM dependency of the number mapping on the MNL in Experiment 1, but evidence for it in Experiment 2, suggesting that the number mapping on the MNL is not fully flexible when certain stimulus sets are used.
Does the strength of the SNARC effect depend on AM in the used range?	*Hypothesis 3:* The SNARC effect is expected to be stronger in the lower than in the higher number ranges.	Two two-sided paired Bayesian *t*-tests of SNARC slopes in lower versus higher number range (one test per experiment). (Note that this test will only be run in case we find at least moderate evidence for a SNARC effect in the lower number range of the respective experiment; see hypothesis 1a, which serves as a manipulation check.)	Finding moderate or even strong evidence for a more negative SNARC slope in one of the two number ranges would indicate that the SNARC effect seems to be stronger in this number range than in the other.	Finding the SNARC effect to be stronger in the lower than in the higher number range would indicate that the spatial mental representation of small numbers is more pronounced than for large numbers (as in scenarios 4, 5 and 6 in electronic supplementary material, figure S1). If the SNARC effect does not differ between number ranges, no evidence can be provided for the strength of the SNARC effect to depend on AM (as in scenarios 1, 2 and 3).	**Experiment 1:** Strong evidence against a difference in SNARC slopes between the ranges with *BF10* = 0.09. **Experiment 2:** Strong evidence for a difference in SNARC slopes between the ranges with BF_10_ = 1271.17. **Summary:** Evidence against AM dependency of the strength of the SNARC effect in Experiment 1, but evidence for it in Experiment 2, suggesting that the spatial mental representation of small numbers is more pronounced than for large numbers when certain stimulus sets are used.

Rationale for the test sensitivity and sampling plan: The most crucial aim of the present study is to find out whether AM dependency of the strength of the SNARC effect exists (*hypothesis 3*). The minimally relevant effect size of *d* = 0.15 was chosen because it corresponds to the SNARC slope difference of 2.99 ms between number ranges (with a pooled SD of 18.34 ms) that was descriptively found but remained non-significant in the original study by Fias *et al.* [8] that we wish to replicate here. Note that due to the lacking report of SD, it is not possible to calculate Cohen’s *d* for the slope difference of 9.2 ms found by Dehaene *et al*. [6]. Importantly, a smaller effect size than *d* = 0.15 would not be meaningful for the SNARC effect (hypothesis 1) or for RM dependency and AM dependency of the number mapping on the MNL (hypothesis 2) either. Similarly, the chosen maximal sample size should be large enough to find at least moderate evidence in case hypotheses 1 and 2 are false.

To reach the desired probability of 0.90 for finding moderate evidence in favour of a true underlying effect (i.e. BF_10_ × > 3) with an effect size of Cohen’s *d* = 0.15 in two-sided Bayesian one-sample *t*-tests or in two-sided Bayesian paired *t*-tests, 800 participants need to be tested in each experiment (for power calculations and sample size estimations, see [37]. The required sample size for finding moderate evidence against a truly absent effect (i.e. BF_10_ < 1/3) for *d* = 0 is only 180. By ensuring our design is sensitive to find evidence for *d* = 0.15, we will be able to detect a slope difference of the size found by Fias *et al.* [8], as predicted by hypothesis 3, and a smaller effect size would not be meaningful for hypotheses 1 and 2 either. However, we will employ the SBF + maxN approach as described by Schönbrodt & Wagenmakers [54]. More precisely, we will first recruit 200 participants and then calculate the BF_10_ for all *t*-tests after each added 20 participants. In case the BF_10_ reach a threshold of 1/3 or of 3 (i.e. moderate evidence for or against hypotheses 1, 2, and 3) before getting to the sample size of 800 participants, we will stop recruiting earlier in the respective experiment.

The key dependent variable was the mean difference between RTs of the right hand minus left hand (dRT), which was calculated for each number separately per participant and per number range. RTs were measured as the time from the onset of the number presentation on the screen until the participant’s response. A potential SNARC effect can be determined by regressing dRTs on the number magnitude [8]. For each participant and for each number range, one regression was calculated. Our first dependent measure was SNARC slopes resulting from the regression of dRTs on number magnitude, which represent the change in relative advantage of right-hand compared to left-hand responses in ms per increase by one in the number magnitude (the more negative the slope, the stronger the SNARC effect). Moreover, we calculated smallest-number intercepts (when RM of the numbers in both ranges was matched, i.e. predicted dRTs for 0 in the 0–5 range versus 4 in the 4–9 range in Experiment 1, and 1 in the 1–5 range (excluding 3) versus 4 in the 4–8 range (excluding 6) in Experiment 2), as well as dRTs for critical numbers that were part of both number ranges (i.e. 4 and 5). An overview of how the tests described in the following helped us distinguish the six scenarios with different number representation shapes, depending on the number mapping on the MNL and the strength of the SNARC effect, is given in electronic supplementary material, figures S1, S2 and table S1.

First, we tested the presence of the SNARC effect on group level in both number ranges separately in each experiment (hypothesis 1). As described in the Introduction, the SNARC effect seems to be stronger in the lower than in the higher number range, resulting in a more negative slope. As the SNARC effect is very robust especially for lower ranges and possibly stronger than in higher ranges (see hypothesis 3), the SNARC effect in lower ranges (hypothesis 1a) was a manipulation check and prerequisite for following investigations (hypotheses 1b, 2 and 3). The obtained SNARC slopes were tested against zero with two-sided Bayesian one-sample *t*-tests in each number range in each experiment. This procedure corresponds to the repeated-measures regressions described by Lorch & Myers [10] and applied to the SNARC effect by Fias *et al*. [8]. It accounts for the within-subject design, where each participant completes trials for each digit in each response-to-key assignment. Although we did not expect the SNARC effect to be reversed at the group level, we preregistered two-sided tests here to stay consistent within this study. Evidence for the SNARC effect in all ranges would replicate findings from the two studies by Dehaene *et al*. [6] and Fias *et al*. [8]. The lack of conclusive evidence as regards the SNARC effect in the lower ranges (hypothesis 1a) with our maximal sample of 800 participants or even evidence against it was highly unlikely in our view. Evidence against the SNARC effect in the higher ranges (hypothesis 1b) combined with evidence for the SNARC effect in the lower ranges (hypothesis 1a) would provide support for AM dependency of the strength of the SNARC effect (hypothesis 3).

Second, to investigate RM dependency of the number mapping on the MNL, we tested whether dRTs for critical numbers (i.e. 4 and 5) differed between the lower and the higher number range (hypothesis 2a) with one two-sided paired Bayesian *t*‐test per number in each experiment. Evidence for a difference would imply that the SNARC effect and the MNL are (at least partly) flexible and adapt to the number range used in a task (as in scenarios 1, 2, 4 and 5 in electronic supplementary material, figures S1 and S2). This would be in line with the literature claiming that numbers 4 and 5 are associated with the right side in the number range from 0 to 5 and with the left side in the number range from 4 to 9. However, this finding would not fully rule out AM dependency. Evidence against a difference would indicate that the SNARC effect and the MNL are AM dependent at least to some degree (as in scenarios 3 and 6 in electronic supplementary material, figures S1 and S2).

Next, to test AM dependency of the number mapping on the MNL, we tested whether the smallest-number intercepts differed between the lower and the higher number range (hypothesis 2b) with one two-sided paired Bayesian *t*‐test in each experiment. Evidence for a difference would lead to the conclusion that small/large numbers are overall shifted to the left/right on the MNL, respectively (as in scenarios 2, 3, 5 and 6 in electronic supplementary material, figures S1 and S2). In other words, this would imply that the SNARC effect and the MNL are not fully RM-dependent. Evidence against a difference would indicate that the SNARC effect and the MNL are at least partly RM-dependent (as in scenarios 1 and 4 in electronic supplementary material, figures S1 and S2).

Third, to investigate AM dependency of the strength of the SNARC effect, we compared SNARC slopes between the number ranges (hypothesis 3) with one two-sided paired Bayesian *t*‐test in each experiment. Evidence for steeper SNARC slopes in the lower than in the higher number range can be interpreted as stronger SNARC effect within (in absolute terms) smaller than larger numbers (as in scenarios 4, 5 and 6 in electronic supplementary material, figures S1 and S2). This result would lead to the conclusion that the spatial mental representation seems to be more pronounced for small than for large numbers. Evidence against such a difference would indicate that the strength of the SNARC effect does not differ between number ranges (as in scenarios 1, 2 and 3 in electronic supplementary material, figures S1 and S2). Once the data were collected, results could be interpreted with the help of electronic supplementary material, table S1 to see which scenario most likely underlies the mental representation of number magnitude.

Additionally, to all effects reported in the unit of interest, we provide effect sizes in terms of Cohen’s *d* for all Bayesian *t*-tests. Effects of *d* ≥ 0.2, *d* ≥ 0.5 or *d* ≥ 0.8 will be interpreted as small, medium or large effect sizes, respectively.

### 2.7. Manipulation checks

To control the data quality in our study, we implemented a seriousness check [56,57,58] as well as a self-assessment of noise, distractions and other difficulties. To make sure that we only analysed trials that reflected mental processes in correctly executed parity judgement, we excluded incorrectly answered trials and trimmed RTs (as described in the data preprocessing pipeline in our Stage-1 Registered Report). Also, we excluded data of participants with less than 75% valid trials to only build our results on participants who understood and followed the task instructions. Moreover, we assessed whether participants complied with the instructions to use their left and right index fingers for the left and right response keys, respectively, and only included their datasets in our analysis if they did so. Then, as a manipulation check, we tested the SNARC effect in the lower number ranges (hypothesis 1a). Importantly, we only proceeded with the testing of other hypotheses because we could find the SNARC effect in the lower number range in both experiments.

## 3. Possible limitations and unexpected outcomes

Finding evidence against the SNARC effect in the lower number ranges (Experiment 1: 0–5; Experiment 2: 1–5 , excluding 3) would have been an unexpected outcome. The SNARC effect in the parity judgement task has been shown in plenty of studies (including online experiments) using different number ranges within the interval from 0 to 9. Our large sample and a high number of repetitions ensured a high probability of finding evidence even for small effects. The presence of the SNARC effect in the lower ranges thus is a manipulation check and prerequisite for further hypothesis tests.

Even though our Experiment 1 was a direct replication of Dehaene *et al*.’s [6] and Fias *et al*.’s [8] study, we decided to use 25 instead of 15 repetitions per experimental cell. First, we thereby increased statistical power and measurement precision [73]; second, we followed methodological recommendations for investigating the SNARC effect [34]; and third, we ensured the comparability with our conceptual replication in Experiment 2. Because of this methodological improvement, our experiment was not a direct replication.

Just as the original two experiments, Experiment 1 had the limitation of the MARC effect being confounded with the SNARC effect because number parity and number magnitude were no orthogonal predictors in the regression model. Therefore, we only calculated the MARC effect for the data resulting from Experiment 2. Moreover, because of the special characteristics of the number 0 regarding its mental representation, including it in the stimulus set might have driven responses in our Experiment 1. However, we tackled these limitations in Experiment 2 by using another stimulus set.

## 4. Results

**Figure 3.**
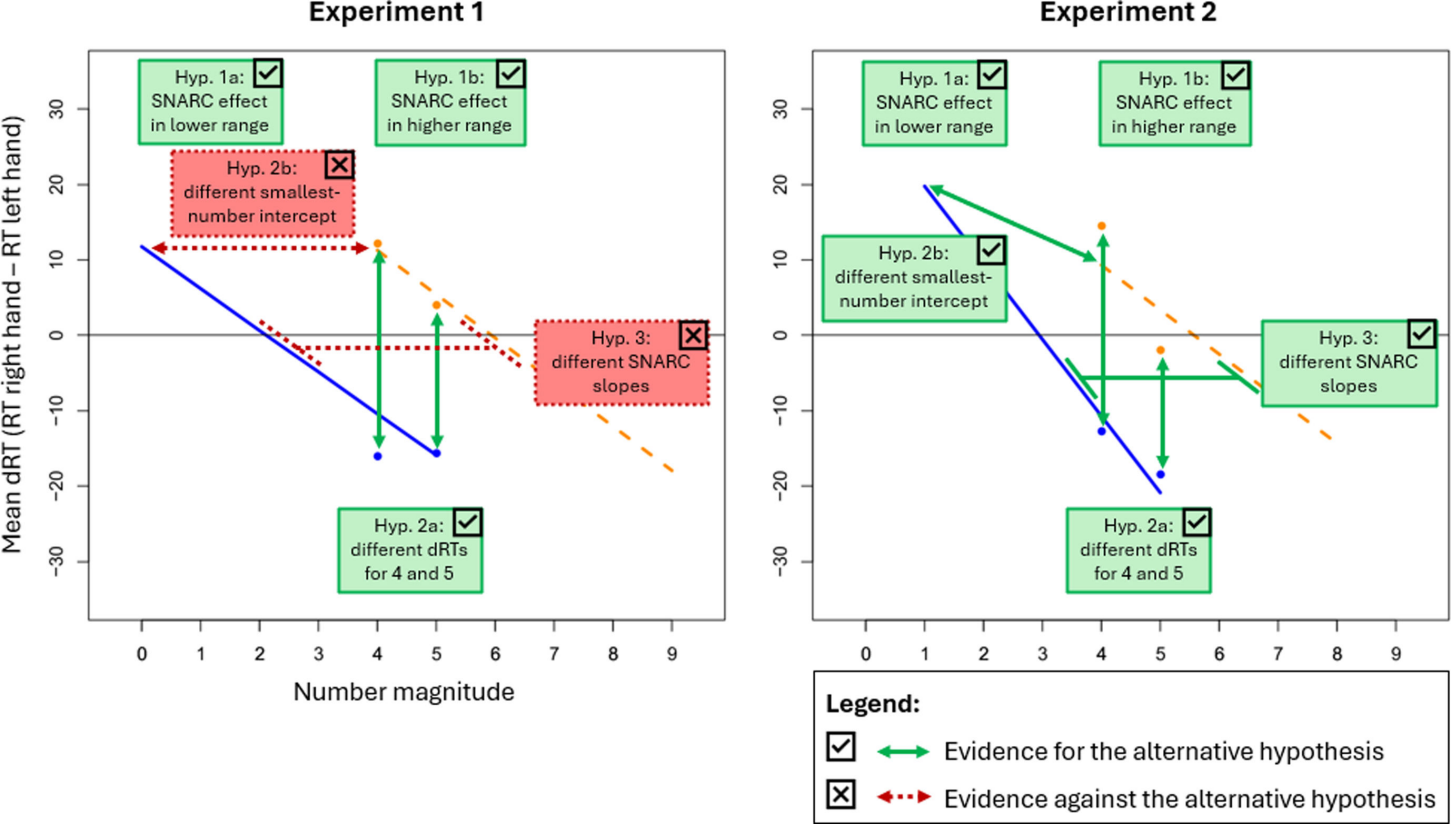
Summary of all tested hypotheses and outcomes in the plot for the linear regression of mean dRTs on number magnitude separately for the lower (blue, solid line) and higher (orange, dashed line) number ranges for experiments 1 (left; see figure 4) and 2 (right; see figure 5). *Note*. The figure only includes the mean dRTs for the critical numbers 4 and 5, which appear in both the lower and the higher number ranges. Hypotheses 1a and 1b were tested with one-sample *t*-tests, whereas hypotheses 2a, 2b and 3 were comparisons tested with paired *t*-tests and are illustrated with two-sided arrows. Green boxes with a solid outline and a checkmark as well as green solid arrows indicate Bayesian evidence for the alternative hypothesis (i.e. BF_10_ > 3). Red boxes with a dotted outline and a cross as well as red dotted arrows indicate Bayesian evidence against the alternative hypothesis (i.e. BF_10_< 1/3).

The data collection as well as the confirmatory data analyses were conducted as described in the peer-reviewed Stage-1 Registered Report, which received in-principle acceptance by the PCI on 3 December 2023 (https://doi.org/10.17605/OSF.IO/AE2C8). Additional data analyses are referred to as exploratory data analyses in the following. All R scripts for data preprocessing and analysis as well as all anonymized datasets can be found at [37]. A Study Design Table was filled in prior to data collection and provides an overview of all research questions, corresponding hypotheses, the targeted sample size and planned analyses with a rationale, the interpretations of potential outcomes and theoretical conclusions (see table 2). It also contains the observed outcomes for both experiments, which are additionally illustrated in figure 3.

**Figure 4.**
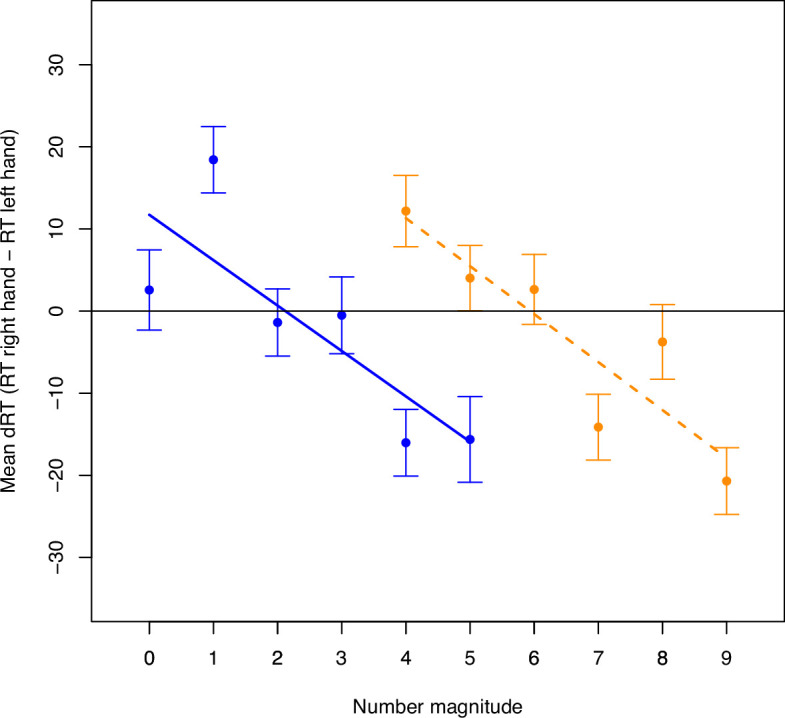
Mean dRTs per number averaged across all trials from all participants in the lower (blue, solid line) and higher (orange, dashed line) number ranges for Experiment 1, with error bars representing ± 1 SE for the respective number and regression lines representing slope estimates for the respective range.

**Figure 5.**
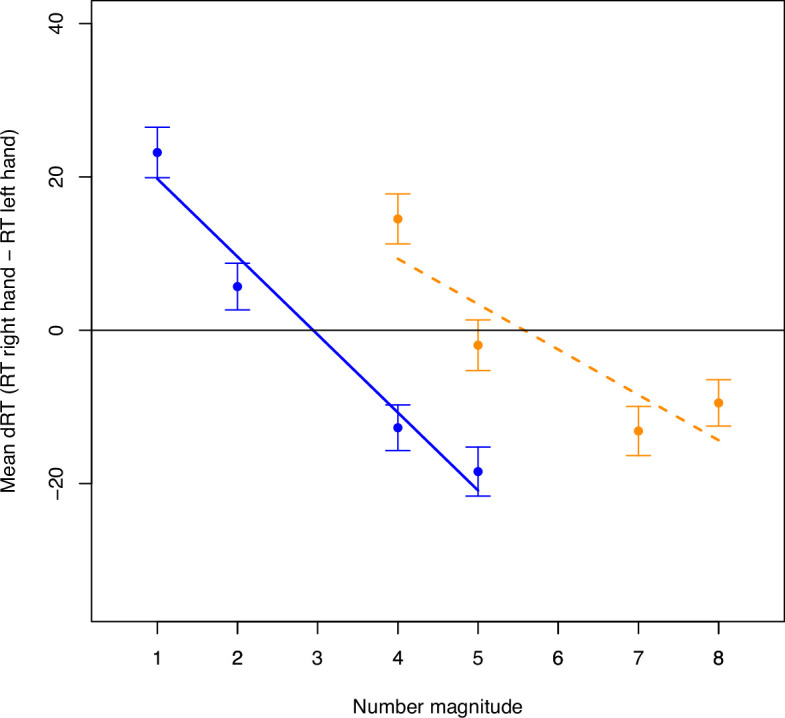
Mean dRTs per number averaged across all trials from all participants in the lower (blue, solid line) and higher (orange, dashed line) number ranges for Experiment 2, with error bars representing ± 1 SE for the respective number and regression lines representing slope estimates for the respective range.

### 4.1. Experiment 1: close replication with 0 to 5 versus 4 to 9

#### 4.1.1. Data preprocessing

Our final recruited sample size according to the SBF + maxN approach was 200. This final sample size refers only to collected individual datasets that were complete, but initially, 208 individuals started participation of which eight did not complete. Of these, one participant was under 18 and two participants were over 40 years old. Three participants did not fully follow the instructions as concerns response-to-key assignment, and seven participants used other fingers than required in the instructions or switched around. The data of these participants were excluded from the analysis in the first preprocessing step. All recruited individuals wanted to seriously participate (i.e. none of them only wanted to look at the experiment), and none of them reported a very/extremely noisy environment or multiple major distractions. In the next preprocessing step, exclusion criteria were applied at the trial level. That is, 0.01% of the trials were excluded due to missing responses, 5.78% due to incorrect responses, 0.12% due to responses faster than 200 ms, 2.17% due to responses slower than 1500 ms and 6.14% in the sequential RT trimming procedure. After these exclusions at the trial level, none of the participants had any empty experimental cells, but 14 participants had less than 75% remaining valid trials and their data were thus excluded from analyses. The remaining data of 173 individuals were analysed. Descriptive self-reported information about these participants is summarized in table 3. All of them used the default response keys D and K. The average RT per number in each range can be found in table 4 and is illustrated in electronic supplementary material, figures S3 and S4. Mean RTs ranged from 525.25 to 567.35 ms and standard errors (SE) ranged from 6.75 to 8.43 ms (note that the descriptively largest SE was observed for number 0, which is in line with previous studies).

**Table 3.** Descriptive self-reported information about the samples in both experiments (*N* = 173 in Experiment 1 and *N* = 255 in Experiment 2 after exclusions).

demographic item	answer	Experiment 1	Experiment 2
gender	woman	79 (45.7%)	121(47.5%)
	man	92 (53.2%)	133 (52.2%)
	diverse	2 (1.2%)	1 (0.4%)
	no answer	0 (0.0%)	0 (0.0%)
age	mean (SD)	30.10 (5.52)	30.20 (5.86)
native language	English	171 (98.8%)	253 (99.2%)
	others	2 (1.2%)	2 (0.8%)
handedness	right-handed	150 (86.7%)	224 (87.8%)
	left-handed	15 (8.7%)	24 (9.4%)
	ambidextrous	8 (4.6%)	7 (2.7%)
finger-counting habit	right-starters	82 (47.4%)	128 (50.2%)
	left-starters	64 (37.0%)	95 (37.3%)
	does not know or no preference	27 (15.6%)	32 (12.5%)
finger-counting stability	always	57 (32.9%)	99 (38.8%)
	mostly	72 (41.6%)	103 (40.4%)
	slightly more often	15 (8.7%)	15 (5.9%)
	does not know or no preference	29 (16.8%)	38 (14.9%)
math skills (0–400)	mean (SD)	240.80 (86.94)	235.80 (94.32)

**Table 4.** Average RT in ms per number in each range (with SEs in parentheses). For plots, see electronic supplementary material, figures S3–S6.

Experiment 1				Experiment 2			
lower range		higher range		lower range		higher range	
number	mean RT (SE RT)	number	mean RT (SE RT)	number	mean RT (SE RT)	number	mean RT (SE RT)
0	566.63 (8.43)	4	525.25 (6.75)	1	517.87 (5.19)	4	513.47 (5.15)
1	567.35 (7.58)	5	544.53 (6.97)	2	512.08 (5.26)	5	519.53 (5.07)
2	538.28 (7.61)	6	540.12 (7.40)	4	514.18 (5.24)	7	508.70 (5.12)
3	547.82 (7.59)	7	529.12 (7.00)	5	522.44 (5.31)	8	501.59 (5.12)
4	539.00 (7.11)	8	529.96 (6.92)	—	—	—	—
5	550.15 (7.52)	9	548.12 (7.28)	—	—	—	—

#### 4.1.2. Confirmatory data analysis

All parameter estimates can be found in table 5.^2^ The average RT for all included trials was 543.44 ms (SD = 88.00 ms). The repeated-measures regressions followed by Bayesian one-sample *t*-tests against zero revealed strong evidence for a SNARC effect in the lower range (i.e. for hypothesis 1a) with BF_10_ = 6956.04 and a slope estimate of −5.53 ms (SD = 14.66 ms, *d* = 0.38), as well as in the higher range (i.e. for hypothesis 1b) with BF_10_ = 2.63 × 10^6^ and a slope estimate of −5.84 ms (SD = 12.33 ms, *d* = 0.47). Hence, the manipulation check confirmed that the manipulation had worked, and the prerequisite for testing all further hypotheses was fulfilled. The SNARC effect is plotted in figure 4 for both ranges (see also figure 3, left panel) and looks similar to scenario 1 illustrated in electronic supplementary material, figure S1 (see electronic supplementary material in ‘PCI Stage 2 Registered Report Materials’ at [37]. Two paired Bayesian *t*-tests revealed strong evidence for differences in dRTs for critical numbers between the ranges and thus for RM dependency of the number mapping on the MNL (i.e. for hypothesis 2a) with BF_10_ = 2.57 × 10^4^ (*d* = 0.40) for number 4 and BF_10_ = 12.86 (*d* = 0.25) for number 5. Another paired Bayesian *t*‐test revealed strong evidence against a difference in smallest-number intercepts between the ranges and thus against AM dependency of the number mapping on the MNL (i.e. against hypothesis 2b) with BF_10_ = 0.09. The last paired Bayesian *t*‐test revealed strong evidence against a difference in SNARC slopes between the ranges and thus against AM dependency of the strength of the SNARC effect (i.e. against hypothesis 3) with BF_10_ = 0.09.

**Table 5.** Parameter estimates for both experiments in the lower and higher number ranges (calculated per participant and averaged across them), with one asterisk indicating moderate evidence and with two asterisks indicating strong evidence for H0 (i.e. no difference between ranges) or for H1 (i.e. difference between ranges).

	Experiment 1			Experiment 2		
	lower	higher	evidence	lower	higher	evidence
SNARC intercept	11.72	34.68	H1**	29.93	33.00	H0*
SNARC slope	−5.53	−5.84	H0*	−10.17	−5.92	H1**
dRT for number 4	−16.03	12.18	H1**	−12.72	14.52	H1**
dRT for number 5	−15.63	4.02	H1*	−18.44	−1.95	H1**
smallest-number intercept	11.72	11.31	H0*	19.76	9.32	H1**
mean-number intercept	−2.09	−3.29	H0*	−0.57	−2.51	H0*
MARC slope	—	—	—	—	10.07	inconcl.

#### 4.1.3. Exploratory data analysis

In addition to the confirmatory data analyses and in order to disentangle the possible scenarios illustrated in electronic supplementary material, figures S1 and S2 and table S1, the mean-number intercepts were compared between ranges. The mean number in the lower range (0–5) was 2.5 with a dRT estimate of −2.09 ms, and the mean number in the higher range (4–9) was 6.5 with a dRT estimate of −3.29 ms. A two-sided paired Bayesian *t*‐test revealed moderate evidence against a difference in mean-number intercepts between the ranges and thus against AM dependency of the number mapping on the MNL with BF_10_ = 0.10.

Moreover, we tested whether there was a correlation between the SNARC slopes in the lower and the higher range. The data revealed moderate evidence against a correlation with BF_10_ = 0.19.

### 4.2. Experiment 2: conceptual replication with 1–5 (excluding 3) versus 4–8 (excluding 6)

#### 4.2.1. Data preprocessing

Our final recruited sample size according to the SBF + maxN approach was 300.^3^ This final sample size refers only to collected individual datasets that were complete, but initially 310 individuals started participation of which 10 did not complete. Of these, two individuals did not want to seriously participate and instead only looked at the experiment, one was over 40 years old, two participants reported a very/extremely noisy environment and three participants reported multiple major distractions. Further, 17 participants did not fully follow the instructions as concerns response-to-key assignment, and 10 participants used other fingers than required in the instructions or switched around. The data of these participants were excluded from the analysis in the first preprocessing step. No participant was under 18 and needed to be removed. In the next preprocessing step, exclusion criteria were applied at the trial level. 0.37% of the trials were excluded due to missing responses, 5.52% due to incorrect responses, 0.47% due to responses faster than 200 ms, 1.22% due to responses slower than 1500 ms and 5.49% in the sequential RT trimming procedure. After these exclusions at the trial level, none of the participants had any empty experimental cells, but 15 participants had less than 75% remaining valid trials, and their data were thus excluded from analyses. The remaining data of 255 individuals were analysed. Descriptive self-reported information about these participants is summarized in table 3. All participants used the default response keys D and K. The average RT per number in each range can be found in table 4 and is illustrated in electronic supplementary material, figures S5 and S6. Mean RTs ranged from 501.59 to 522.44 ms and SEs ranged from 5.07 to 5.31 ms.

#### 4.2.2. Confirmatory data analysis

All parameter estimates can be found in table 5. The average RT for all included trials was 512.77 ms (SD = 74.46 ms) (table 5). The repeated-measures regressions followed by Bayesian one-sample *t*-tests against zero revealed strong evidence for a SNARC effect in the lower range (i.e. for hypothesis 1a) with BF_10_ = 1.61 × 10^21^ and a slope estimate of −10.17 ms (SD = 14.32 ms, *d* = 0.71), as well as in the higher range (i.e. for hypothesis 1b) with BF_10_ = 1.38 × 10^12^ and a slope estimate of −5.92 ms (SD = 14.32 ms, *d* = 0.52). Hence, the manipulation check worked (i.e. evidence for hypothesis 1a was obtained), and the prerequisite for testing all further hypotheses was fulfilled. The SNARC effect is plotted in figure 5 for both ranges (see also figure 3, right panel) and seems to correspond to scenario 5 presented in electronic supplementary material, figure S2. Two paired Bayesian *t*-tests revealed strong evidence for differences in dRTs for critical numbers between the ranges and thus for RM dependency of the number mapping on the MNL (i.e. for hypothesis 2a) with BF_10_ = 6.64 × 10^7^ (*d* = 0.42) for number 4 and BF_10_ = 64.64 (*d* = 0.24) for number 5. Another paired Bayesian *t*‐test revealed strong evidence for a difference in smallest-number intercepts between the ranges and thus for AM dependency of the number mapping on the MNL (i.e. for hypothesis 2b) with BF_10_ = 546.98 (*d* = 0.27). The last paired Bayesian *t*‐test revealed strong evidence for a difference in SNARC slopes between the ranges and thus for AM dependency of the strength of the SNARC effect (i.e. for hypothesis 3) with BF_10_ = 1271.17 (*d* = 0.28).^4^

#### 4.2.3. Exploratory data analysis

As for Experiment 1, to disentangle the possible scenarios illustrated in electronic supplementary material, figures S1 and S2 and table S1 in Experiment 2, the mean-number intercepts were compared between ranges. The mean number in the lower range (1–5, excluding 3) was 3 with a dRT estimate of −0.57 ms, and the mean number in the higher range (4–8, excluding 6) was 6 with a dRT estimate of −2.51 ms. A two-sided paired Bayesian *t*‐test revealed moderate evidence against a difference in mean-number intercepts between the ranges and thus against AM dependency of the number mapping on the MNL with BF_10_ = 0.18.

As in Experiment 1, we tested whether there was a correlation between the SNARC slopes in the lower and the higher range. In contrast to the data of Experiment 1, the data of Experiment 2 revealed strong evidence for a moderate correlation with an estimate of *r* = 0.34 and BF_10_ = 5.22 × 10^5^.

In contrast to Experiment 1, number parity and number magnitude were orthogonal in Experiment 2 (i.e. the mean number magnitude was equal for odd and even numbers in each range). Therefore, we were also able to test the MARC effect in Experiment 2. A two-sided Bayesian one-sample *t*‐test of the MARC slopes against zero revealed moderate evidence against a MARC effect in the lower range with BF_10_ = 0.16 and inconclusive evidence regarding a MARC effect in the higher range with BF_10_ = 0.51.

## 5. Discussion

The goal of the present study was to determine the degree of flexibility of SNAs. More precisely, we wanted to find out whether the SNARC effect is entirely flexible and depends only on relative magnitude (RM dependency) or whether it is less flexible than previously assumed and also depends on absolute magnitude (AM dependency). Importantly, the concepts of RM dependency versus AM dependency can be differentiated in the following two ways: (i) the number mapping on the MNL (e.g. dRT for number 4) can be RM-dependent, AM-dependent or both, and (ii) the strength of the SNARC effect in terms of the relative increase of right-hand advantage per increase in magnitude (i.e. the SNARC slope) can be AM dependent or not. To summarize, the aim of the study was to determine whether SNAs operate on fixed and flexible number representations simultaneously.

### 5.1. RM dependency and AM dependency of SNAs

In the two seminal studies by Dehaene *et al*. [6] (Experiment 3) and by Fias *et al*. [8] (Experiment 1), numbers 4 and 5 were associated with the right when presented in the range from 0 to 5, but with the left when presented in the range from 4 to 9. These results are often quoted in the literature as evidence for pure RM dependency, although the studies were underpowered for small effects and descriptive data suggests AM dependency as well. In Experiment 1, which was run online with the same stimulus sets as in the two original studies, we replicated the findings from the original studies. Specifically, we found strong evidence for a small-sized SNARC effect in the lower (average slope of −5.53) and higher (average slope of −5.84) range separately (hypothesis 1), as well as for small dRT differences for critical numbers (i.e. 4 and 5) between the ranges (hypothesis 2a), in line with the original studies. Moreover, we observed strong evidence against smallest-number intercept (hypothesis 2b) and SNARC slope differences (hypothesis 3), as well as moderate evidence against mean-number intercept differences (exploratory analysis) between the lower and the higher range. To conclude, the results from our investigation are entirely in line with the results from the original studies, and in fact, without further consideration (see below), they seem to support the conclusion of full RM dependency drawn by Dehaene *et al*. [6] and Fias *et al*. [8]: whereas their data hinted descriptively towards AM dependency as pointed out in the Introduction, we even observed moderate evidence against it.

Crucially, the second experiment in the present study yielded different results. In Experiment 2, we ran a conceptual online replication considering recent advances in SNARC research with the number ranges from 1 to 5 (excluding 3) and from 4 to 8 (excluding 6). As opposed to the original stimulus set, this modified stimulus set avoids potential confounds with the MARC effect thanks to the equal number of odd and even numbers in the original stimulus set and parity being orthogonal to magnitude. Also, number 0 was excluded because it has special properties and deviates in its dRT pattern from all other numbers (see [41] for detailed analysis). In line with the literature, the dRT pattern for number 0 observed in the current study did not align well with the regression line, and the RT variation was descriptively larger for number 0 than for all other numbers. Therefore, one would not want to base a general statement about the flexibility of the number line on number 0, because the relatively low dRT estimate for 0 might considerably attenuate the SNARC effect in the lower range. However, these two number ranges used in Experiment 2 still include the same critical numbers that are part of both number ranges in the original studies, namely 4 and 5. As in Experiment 1, we found strong evidence for the SNARC effect in the lower (average slope of −10.17) and higher (average slope of −5.92) range separately (hypothesis 1). Note that in contrast to the small effect size in both ranges in Experiment 1 (*d* = 0.38 and *d* = 0.47), the effect size was medium in both ranges in Experiment 2 (*d* = 0.71 and *d* = 0.52). Thus, our results support the claim that the inclusion of number 0 in the stimulus set or a potential confound with the MARC effect due to an unequal number of odd and even numbers which might have decreased the SNARC effect in the seminal studies by Dehaene *et al*. [6] and Fias *et al*. [8]. Moreover, as in Experiment 1, strong evidence was found for dRT differences for critical numbers (i.e. 4 and 5) between ranges (hypothesis 2a). Further, as in Experiment 1, moderate evidence was found against mean-number intercept differences between ranges (exploratory analysis). However, the support for RM dependency is not the entire story.

Importantly, the data revealed strong evidence for differences in smallest-number intercept (hypothesis 2b) and SNARC slopes (hypothesis 3) between the ranges. These differences indicating AM dependency are only small (*d* = 0.27 and *d* = 0.28 for hypotheses 2b and 3, respectively), but so are the differences indicating RM dependency (*d* = 0.42 and *d* = 0.24 for hypothesis 2a). Hence, in contrast to the pure RM dependency observed in Experiment 1, the results from Experiment 2 suggest AM dependency both for (i) the number mapping on the MNL and (ii) the strength of the SNARC effect (in line with scenario 5 in the electronic supplementary material).

How can these results be reconciled with Experiment 1, where we observed evidence against AM dependency? As outlined above, we conducted Experiment 2 (among other reasons) to see whether 0 drives results for the number ranges from 0 to 5 and from 4 to 9 used in the original experiments by Dehaene *et al*. [6] and Fias *et al*. [8]. Therefore, we reanalysed the data in an exploratory way without 0. Note this is not the same as if the experiment had been run without number 0. It was part of the stimulus set, even when it was not analysed later, and the number range used in the experiment might still influence results. When excluding number 0 from the stimuli set in Experiment 1 post hoc,^1^ the evidence against AM dependency disappeared. Instead, the evidence was now inconclusive regarding smallest-number intercept differences (hypothesis 2b) and SNARC slope differences (hypothesis 3). Since number 0 was still part of the range of Experiment 1, we did not expect the same results as in Experiment 2, which was run without 0. However, when the results of Experiment 1 are analysed without 0, there is at least no conflicting evidence anymore. This change suggests that the inclusion of 0 in some range plays a major role for the eventual outcome.

Notably, the dRT was also rather high for number 1 in both experiments and pulled the regression line upwards. That is, number 1 seems to be more strongly associated with the left than what would be predicted based on the regression slope alone. This observation fits with the results from our two previously conducted colour judgement tasks, which do not require semantic number processing at all [32]. Number 1 seemed to be strongly associated with the left in these experiments as well, providing further support for AM dependency of the MNL.

In the present study, Bayesian analyses allowed us to interpret and quantify evidence for the null hypotheses. Moreover, these findings can be considered as trustworthy, because the sample sizes were large enough (173 datasets analysed out of 200 recruited participants in Experiment 1 and 255 datasets analysed out of 300 recruited participants in Experiment 2). Thanks to the SBF + maxN approach, an optimally sized sample was recruited in each experiment. These samples were much larger than in the original studies, where a difference between number ranges might just have stayed undetected due to lacking statistical power.

To sum up, although the picture is blurred by methodological issues, especially the inclusion of number 0 in one but not in the other range, in the original studies and in our Experiment 1, the findings from Experiment 2 together with the reanalysis of Experiment 1 without 0 seem to suggest that there is not the ‘one and only SNARC’. The spatial mapping of numerical magnitude onto space seems not to be fully flexible and dependent on the used range. Null effects of AM can only be found when number 0 is included. We therefore conclude that the SNARC effect seems to operate on multiple number representations and on multiple spatial reference frames simultaneously, namely on both flexible and absolute ones.

### 5.2. RM dependency and AM dependency from a theoretical point of view

As outlined in the Introduction, different predictions regarding the SNARC effect’s flexibility can be derived from the models that have been proposed to account for the origin of the SNARC effect. The working memory account [28,29] postulates that the SNARC effect is constructed during task execution. In the present study, the currently used stimulus set (i.e. the lower or the higher number range) was stored in working memory, which was reflected by the differential patterns for critical numbers 4 and 5. Namely, when these critical numbers were the highest (i.e. in the lower ranges in both experiments), they were associated with the right, whereas they were associated with the left when they were the lowest (i.e. in the higher ranges in both experiments). This observed RM dependency is clearly in line with the working memory account. In contrast, although the MNL [6] is claimed to dynamically adapt to task demands as well, such that zooming in and out is possible [17], it can be considered as a mental representation in long-term memory. Similarly, the verbal–spatial coding account [25] and polarity correspondence account [26] postulate number representations stored in long-term memory. Crucially, long-term representations hardly justify the SNARC effect’s flexibility [15,27]. Instead, they are in line with the AM dependency observed in Experiment 2, reflected by (i) the number mapping on the MNL in terms of smallest-number intercept differences between ranges and (ii) the degree of spatialization in terms of differences between ranges regarding the strength of the SNARC effect. A potential explanation for this AM dependency is that small numbers are more frequently used than large numbers, which might lead to a more fixed and stronger spatial mental representation. Importantly, RM dependency and AM dependency can coexist (in line with [14,27,30]) and multiple spatial reference frames can be activated simultaneously [31].

An MNL account, in which SNAs are partly flexible (aka zoomed in) and partly fixed (i.e. always more left for absolutely smaller numbers), can also explain the current data (see [30]). However, any account, which wishes to explain the current data, needs a fixed and a flexible component. Any fully flexible account is in our view not consistent with these data.

### 5.3. Correlation of the SNARC effect between ranges

Apart from the SNARC effect at the group level, one can also investigate the phenomenon at the individual level [47,74]. Assessing parity judgement in both stimulus ranges in a within-subjects design permits to investigate the correlation of SNARC effects between ranges (exploratory analyses), which was not tested in the original studies. Surprisingly, Experiment 1 revealed moderate evidence against a correlation of SNARC slopes between ranges, whereas Experiment 2 revealed strong evidence for a moderate correlation, with an estimate of *r* = 0.34. A hypothetical explanation could be that the inclusion of number 0 in the stimulus set of Experiment 1 might have blurred a true underlying correlation.^5^ That is, number 0 showed descriptively stronger variations in RTs than other numbers did, which is in line with the literature (see figure 4 in Nuerk *et al*. [41]), thus introducing a large error term for the dRT. The dRT for number 0 is rather uncorrelated with dRTs for other numbers [41], so slopes built on a stimulus set including 0 are also more likely to be uncorrelated with other slopes built on stimulus sets excluding 0. This large variation in turn results in increased noise for the SNARC slopes in the lower number range in Experiment 1. To summarize, our results from Experiment 2 show a positive relationship between the strength of the spatial mapping in small and large numbers within participants.

## 6. Conclusion

The current study demonstrates that the spatial mental representation of numbers is not entirely flexible. Mental spatialization can be adapted to the context and depends largely on RM, but at the same time, it is also influenced by AM. RM and AM play a role in (i) the association of specific numbers with horizontal directional space, such that numbers that are small in relative or absolute terms are associated with the left, whereas numbers that are large in relative terms are associated with the right. At the same time, RM and AM play a role in (ii) the strength of the spatialization, such that the association of small with left and large with right is stronger within a lower than within a higher number range. To conclude, the spatial representation of number magnitude seems to be partly flexible and partly fixed.

## Data Availability

Demo version for Experiment 1 is available at https://exp.wextor.eu/esnarc/flex/?demo&e1 and for Experiment 2 at https://exp.wextor.eu/esnarc/flex/?demo&e2). All R scripts for data preprocessing and data analysis, as well as all anonymized datasets are available online at [37]. Supplementary material is available online [75].

## Footnotes

^1^
Note that Prolific claims that only one participation from each household is allowed per study. Nevertheless, we received two different complete individual datasets with the same User-Agent, IP address and Prolific ID. The analyses reported in the current paper are based on both datasets. However, we reran the analyses while excluding the second of these two individual datasets and also both of these individual datasets. Importantly, apart from small changes in the decimals of the obtained Bayes factors, the results and interpretations did not change substantially in either of the two reanalyses.

^2^
When excluding number 0 from the lower and number 4 from the higher range in the analyses, the results of the confirmatory data analysis did not change substantially for hypothesis 1a with BF_10_ = 2.09 × 10 and for hypothesis 1b with BF_10_ = 6.65 × 10, and naturally remained the same for the dRT of number 5 in hypothesis 2a. However, for hypotheses 2b and 3, the exclusion of number 0 from the lower and number 4 from the higher range led to inconclusive evidence regarding AM dependency of the number mapping on the MNL and of the strength of the SNARC effect, with BF_10_ = 2.56 and BF_10_ = 0.50, respectively. This stands in contrast with moderate evidence against AM dependency found when including number 0 in the lower and number 4 in the higher range.

^3^
Note that due to a computing mistake, data collection was only stopped with 300 participants, although the stopping criterion for the SBF + maxN procedure (i.e. at least moderate Bayesian evidence for or against each hypothesis) was already reached with 200 participants. Importantly, using data from 300 instead of only 200 participants did not change any results substantially and mostly made the Bayesian evidence stronger (see next footnote).

^4^
If data collection had been stopped with a sample size of 200 participants, results would have been similar with BF_10_ = 4.13 × 10 (*d* = 0.70) for hypothesis 1a, BF_10_ = 2.34 × 10 (*d* = 0.57) for hypothesis 1b, BF_10_ = 5563.96 (*d* = 0.37) for number 4 and *BF_10_* = 75.13 (*d* = 0.29) for number 5 for hypothesis 2a, BF_10_ = 33.56 (*d* = 0.27) for hypothesis 2b and BF_10_ = 19.46 (*d* = 0.26) for hypothesis 3. Thus, evidence would also have been strong and in favour of each hypothesis with 200 participants, just like with 300 participants.

^5^
Excluding number 0 from the lower and number 4 from the higher range in Experiment 1 in the analysis did not affect the results regarding the correlation: moderate evidence against a correlation was found, with BF_10_ = 0.22. Note that this does not contradict the strong evidence for a correlation in Experiment 2, because number 0 was part of the stimulus set when running Experiment 1 and might have undermined the potential correlation.
